# Identification and QTL mapping of resistance to Turnip yellows virus (TuYV) in oilseed rape, *Brassica napus*

**DOI:** 10.1007/s00122-019-03469-z

**Published:** 2019-11-05

**Authors:** Dieter Hackenberg, Elvis Asare-Bediako, Adam Baker, Peter Walley, Carol Jenner, Shannon Greer, Lawrence Bramham, Jacqueline Batley, David Edwards, Regine Delourme, Guy Barker, Graham Teakle, John Walsh

**Affiliations:** 1 School of Life Sciences, University of Warwick, Wellesbourne Campus, Warwick, CV35 9EF UK; 2 Functional and Comparative Genomics, Institute of Integrative Biology, University of Liverpool, Liverpool, L69 7ZB UK; 3 School of Biological Sciences, Institute of Agriculture, The University of Western Australia, Crawley, WA Australia; 4 IGEPP, INRA, Agrocampus Ouest, Université Rennes, Le Rheu, France

## Abstract

**Key message:**

Partially dominant resistance to Turnip yellows virus associated with one major QTL was identified in the natural allotetraploid oilseed rape cultivar Yudal.

**Abstract:**

Turnip yellows virus (TuYV) is transmitted by the peach-potato aphid (*Myzus persicae*) and causes severe yield losses in commercial oilseed rape crops (*Brassica napus*). There is currently only one genetic resource for resistance to TuYV available in brassica, which was identified in the re-synthesised *B. napus* line ‘R54’. In our study, 27 mostly homozygous *B. napus* accessions, either doubled-haploid (DH) or inbred lines, representing a diverse subset of the *B. napus* genepool, were screened for TuYV resistance/susceptibility. Partial resistance to TuYV was identified in the Korean spring oilseed rape, *B. napus* variety Yudal, whilst the dwarf French winter oilseed rape line Darmor-*bzh* was susceptible. QTL mapping using the established Darmor-*bzh* × Yudal DH mapping population (DYDH) revealed one major QTL explaining 36% and 18% of the phenotypic variation in two independent experiments. A DYDH line was crossed to Yudal, and reciprocal backcross (BC_1_) populations from the F_1_ with either the susceptible or resistant parent revealed the dominant inheritance of the TuYV resistance. The QTL on ChrA04 was verified in the segregating BC_1_ population. A second minor QTL on ChrC05 was identified in one of the two DYDH experiments, and it was not observed in the BC_1_ population. The TuYV resistance QTL in ‘R54’ is within the QTL interval on Chr A04 of Yudal; however, the markers co-segregating with the ‘R54’ resistance are not conserved in Yudal, suggesting an independent origin of the TuYV resistances. This is the first report of the QTL mapping of TuYV resistance in natural *B. napus*.

**Electronic supplementary material:**

The online version of this article (10.1007/s00122-019-03469-z) contains supplementary material, which is available to authorized users.

## Introduction

Oilseed rape (OSR; *Brassica napus*; genome AACC, 2*n* = 38) is the second most important oilseed crop after soybean in the world; however, in Europe, OSR is the major source of vegetable oil, oilseed meals and biodiesel (Carré and Pouzet 2014). Annual global production of OSR has doubled since 2000 (FAO 2018) and reached 76.2 million tonnes in 2017, which is equivalent to 14% of the worldwide oilseed crop production. OSR and vegetable brassicas, however, are not reaching their full yield potential in the UK. The average yield for oilseed rape in the UK (3.4 tonne/ha) (DEFRA 2018) is clearly below the estimated potential yields of current cultivars (6.5–7 tonne/ha) (Berry and Spink 2006). Turnip yellows virus (TuYV), formerly known as Beet western yellows virus (BWYV), is considered to be a major contributor to this shortfall (Stevens et al. 2008). TuYV is a *Polerovirus* (*Luteoviridae* family) transmitted by aphids in a persistent, non-circulative manner. The main vector of TuYV is the peach-potato aphid *Myzus persicae* and annual sampling has shown that up to 72% of winged *M. persicae* carried TuYV (Stevens et al. 2008). TuYV transmission by *M. persicae* is highly efficient with transmission rates of over 90% (Schliephake et al. 2000). Surveys in the UK revealed that 42–70% of oilseed rape crops were infected with TuYV (Hardwick et al. 1994). Incidences of 10–85% have been recorded within crops (Hardwick et al. 1994; Hill et al. 1989; Jay et al. 1999; Walsh et al. 1989). TuYV is seen as the most widespread and common disease in oilseed rape in Europe. Symptoms of TuYV infection are often reminiscent of abiotic stress, particularly nutrient deficiency, and can include reddening of leaves and stunted growth. In addition, TuYV infection has been shown to reduce seed yield in single OSR plants by 40–50% (Schroeder 1994) and cause yield losses in OSR crops of 11–46% (Graichen and Schliephake 1999; Jay et al. 1999; Jones et al. 2007). In the past, the most common strategy to control TuYV has been the use of chemical measures against the vector, in particular insecticide (neonicotinoid)-treated seeds, but most of these treatments are now banned for OSR in the EU, emphasising the need for alternative control measures such as natural plant resistance.

The only characterised genetic source of TuYV resistance in brassica to date is the re-synthesised *B. napus* line ‘R54’. This has been incorporated into several commercial OSR varieties. It is associated with a single dominant QTL on ChrA04 and provides incomplete resistance to TuYV (Graichen 1994; Juergens et al. 2010). The expression of the ‘R54’ resistance has been reported to be influenced by environmental factors such as temperature. Elevated ambient temperatures are thought to affect the development of high TuYV titres in infected plants, diminish TuYV resistance or even lead to its breakdown (Dreyer et al. 2001). A general effect of high temperature promoting stress and increasing TuYV susceptibility in OSR was reported previously (Graichen 1998). Increased TuYV incidence as a consequence of mild winters resulting in higher vector activity would indicate that global warming is likely to exacerbate yield losses of OSR crops caused by TuYV infection. Additional genetic resources for TuYV resistance breeding are gaining importance in commercial OSR production.

To identify new genetic sources of TuYV resistance, a *B. napus* diversity set representing a structured sampling of diversity across the *B. napus* genepool including doubled-haploid and inbred lines of winter OSR, spring OSR, kale and swede and re-synthesised *B. napus*, from different regions of the world were screened for TuYV susceptibility. The objective of this study was to identify QTLs associated with TuYV resistance and validate the corresponding QTLs in a BC_1_ generation.

## Materials and methods

### Plant material

Variation in TuYV susceptibility was studied in a diverse subset of 27 accessions from the *B. napus* diversity set developed at Warwick Crop Centre (Table 1). For QTL analysis of TuYV resistance in *B. napus* cultivar Yudal, two mapping populations were used. Firstly, the doubled-haploid (DH) population DYDH (Darmor-*bzh* x Yudal) of 120 individuals derived from the cross of French winter oilseed rape Darmor-*bzh* line with the Korean spring oilseed rape cultivar Yudal (Delourme et al. 2006). Secondly, to verify QTLs identified in the DYDH population, a backcross (BC_1_) population was produced by crossing a TuYV-susceptible DYDH line (DYDH130) to a Yudal plant to produce an F_1_ population (Yudal × DYDH130). Thirteen F_1_ plants were challenged with TuYV and tested for TuYV titre. A partially TuYV-resistant F_1_ plant was crossed with the susceptible parent Darmor-*bzh* to produce a segregating BC_1_ population (Darmor-*bzh* × [Yudal × DYDH130]; see Fig. S1 for a summary of the crossing strategy). To further assess dominance/recessivity of the TuYV resistance, a second BC_1_ population was generated by crossing a different partially TuYV-resistant F_1_ plant with Yudal ([Yudal × DYDH130] × Yudal). All plants were cultivated in Levington’s M2 peat compost in an insect-proof, air-conditioned glasshouse at 18 °C. The commercial oilseed rape variety ‘Caletta’ (Semundo Ltd., now Senova, Cambridge, UK) possesses the TuYV resistance originating from the re-synthesised *B. napus* line ‘R54’ and was used as a control for genotyping corresponding co-segregating markers (Juergens et al. 2010).

**Table 1:** *Brassica napus* accessions tested for resistance/susceptibility to Turnip yellows virus (TuYV) infection

Accession name	Crop type	Genetic status	Country of origin
Apex	Winter oilseed rape	S_1_	Denmark
Bienvenu DH4	Winter oilseed rape	DH	France
Brauner Schnittkohl DH2	Siberian kale	DH	Germany
Bronowski DH1	Spring forage rape	DH	Poland
Canard DH13	Winter forage rape	DH	UK
Capricorn DH1	Winter oilseed rape	DH	UK
Couve Nabica DH2	Couve nabica	DH	Portugal
Darmor-*bzh*	Winter oilseed rape	Inbred	France
Dwarf Essex DH4	Forage rape	DH	UK
English Giant DH1	Winter fodder rape	DH	UK
Hanna DH1	Spring oilseed rape	DH	Sweden
Jet Neuf DH1	Winter oilseed rape	DH	France
Judzae DH2	Swede landrace	DH	South Korea
Major DH	Winter oilseed rape	DH	France
Moana, Moana rape DH3	Fodder rape	DH	New Zealand
Monty-028DH	Spring OSR	DH	Australia
Ningyou 7	Winter oilseed rape	DH	China
Q100	Synthetic	DH	**–**
Rafal DH1	Winter oilseed rape	DH	France
Sarepta DH1	Winter OSR	DH	France
Sensation NZ DH4	Swede	DH	New Zealand
Stellar DH	Spring oilseed rape	DH	Canada
Tapidor DH	Winter oilseed rape	DH	France
Victor	Winter oilseed rape	unspecified	Sweden
Vige DH1	Swede	DH	Norway
Westar DH10	Spring oilseed rape	DH	Canada
Yudal	Spring oilseed rape	DH	South Korea

### Phenotyping

The TuYV isolate used for phenotyping originated from OSR in Suffolk, UK (Patron 1999) and was maintained in OSR cv. ‘Mikado’ in an insectary under 16-h photoperiod at 20 ± 2 °C by serial transmission using *M. persicae* (Mp1 s clone). *M. persicae* carrying the TuYV isolate were used to challenge plants at the 3–4 true leaf stage (3 weeks post sowing). One leaf segment from either a TuYV-infected, or from an uninfected oilseed rape plant, carrying 8–10 *M. persicae*, was placed on individual plants for TuYV challenge or as control, respectively. After a period of 7 days, the aphids were killed using insecticide sprays 0.4 ml/L Lambda-cyhalothrin (Hallmark Zeon, Syngenta, Fulbourn, UK) and 0.75 g/L Pymetrozine (Plenum W.G., Syngenta, Fulbourn, UK). Two DYDH populations (SP1 and SP2; 115 DYDH lines per experiment) and two BC_1_ populations (200 plants per population) were challenged with TuYV. The relative levels of TuYV in plants were determined 6 weeks post-TuYV challenge by triple antibody sandwich enzyme-linked assay (TAS-ELISA) and absorbances (A_405nm_) were recorded as described previously (Hunter et al. 2002).

### Data analyses

Analysis of variance and means separation using least significant differences (LSD) for TAS-ELISA A_405nm_ values were carried out using GenStat Release version 12.1 (Payne et al. 2009). One-way ANOVA tests were performed to identify significant divergence between the *B. napus* accessions, D’Agostino–Pearson test for normality (*α* = 0.05) was applied to assess distribution of ELISA absorbance values in DYDH and BC_1_ populations used in QTL analysis experiments and non-parametric Spearman correlation was calculated to assess the reproducibility of the resistance tests of both DYDH experiments (SP1 and SP2), using GraphPad Prism version 8.1.0 (GraphPad Software, San Diego, California USA).

### QTL analysis

One-hundred and twenty DH lines of the DYDH population were genotyped using a Brassica 20 K single nucleotide polymorphism (SNP) array (Chalhoub et al. 2014) and a genetic map was estimated using JoinMap 4 (van Ooijen 2006). For the BC_1_ from the Darmor-*bzh* × F_1_ cross, DNA was isolated from 107 individuals by LGC Genomics (Hoddesdon, UK) and genotyped using the Illumina Brassica 60 K Infinium SNP array at the University of Western Australia. QTL associated with TuYV resistance were identified using R/qtl (Broman et al. 2003). The distribution of some ELISA values significantly deviated from normality and relevant ELISA values were transformed appropriately. If transformation did not achieve a normal distribution, single QTL analysis (scanone) was performed using a non-parametric algorithm (Kruglyak and Lander 1995). For all other datasets, single QTL (scanone) and two-dimensional QTL (scantwo) analyses were performed using the Haley–Knott regression (Haley and Knott 1992). Genome-wide LOD significance (*α* < 0.05) was determined by permutation test with 10,000 permutations for scanone and 1000 for scantwo. For multiple QTL modelling (MQM), a stepwise forward/backward search algorithm (stepwiseqtl; max. qtl = 4) was performed to identify the QTL model of maximum penalised LOD score (Broman and Sen 2010; Manichaikul et al. 2009). Confidence intervals (CI) of 1.5 LOD were calculated and extended to the next adjacent marker to define QTL intervals.

### Genomic DNA extraction and PCR genotyping

Genomic DNA was extracted from leaf tissue of *B. napus* accessions Yudal, Darmor-*bzh* and from ‘Caletta’ seeds according to the method published previously (Dellaporta et al. 1983). PCR amplification of the ‘R54’ TuYV resistance co-segregating markers was performed using specific primers for marker STS3e32m50-447-320 (forward: 5′-GATCCGTTTGGGTCTTGGTA-3′; reverse: 5′-TTGATGTGAAACGCACATTG-3′) and STS1e31m48-437 (forward: 5′-ATCGGACATTGGTCAGGTTC-3′; reverse 5′-CATACCCCACTGGTTCTTGG-3′) as described previously (Juergens et al. 2010) and amplicons were sequenced by Sanger sequencing.

## Results

### TuYV susceptibility in diverse *B. napus* accessions

**Fig. 1:**
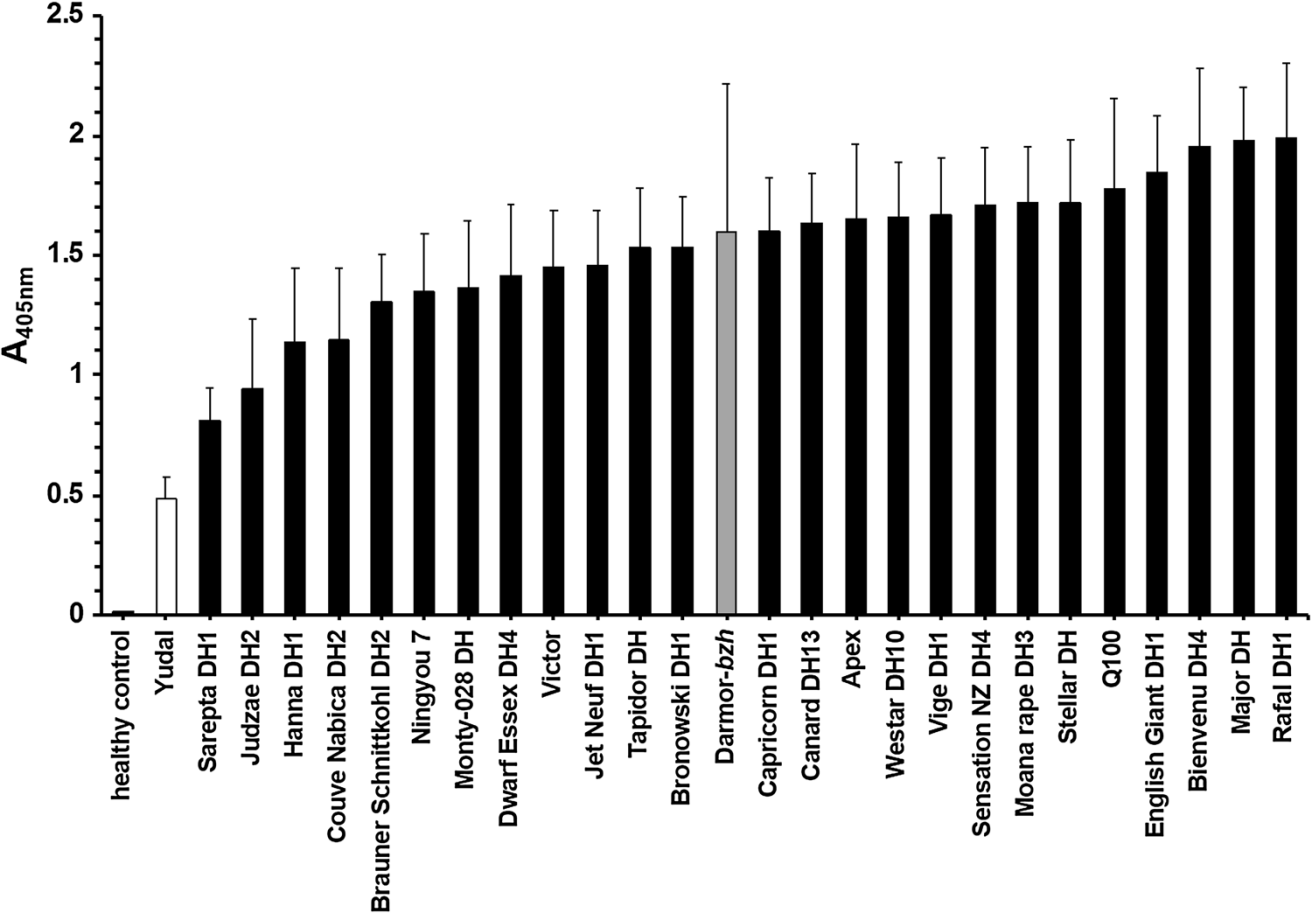
Turnip yellows virus (TuYV) challenge of *Brassica napus* accessions. Means and standard error of A_405nm_ determined via TAS-ELISA. Yudal and Darmor-*bzh* are indicated in white and grey, respectively

The susceptibility of *B. napus* plant lines to TuYV varied based on virus titres determined 6 weeks after infection using TAS-ELISA. The mean virus titre (absorbance, A_405nm_) for the different accessions ranged from 0.485 to 1.985 (Fig. 1) with significant differences [one-way ANOVA test; *F* (26,183) = 1.895, *p* = 0.0082]. All *B. napus* accessions showed significantly higher mean absorbance values than the mean absorbance of the healthy control plants (A_405nm_ = 0.014 ± 0.01), indicating that all tested accessions were infected by TuYV. The highest virus titre was detected in Rafal DH1 (A_405nm_ ± SE = 1.985 ± 0.318), whilst Yudal had the lowest virus titre (A_405nm_ ± SE = 0.485 ± 0.09). In total, 24 of the 27 *B. napus* accessions developed virus titres of > 50% of the maximum virus concentration measured in Rafal DH1, two accessions showed infection between 25 and 50% of the maximum and only Yudal was < 25% of the maximum. Although not showing complete resistance, Yudal (A_405nm_ = 0.485) had a significantly lower virus titre than all other lines (LSD for comparing mean absorbance values between accessions was 0.411 at *df* = 163, *P* < 0.05), except Sarepta (A_405nm_ = 0.812). The TuYV titre of challenged Darmor-*bzh* was > 3 times that in Yudal (A_405nm_ ± SE = 1.595 ± 0.619 vs 0.485 ± 0.09), showing that Darmor-*bzh* is significantly more susceptible to TuYV than Yudal.

### TuYV resistance phenotyping of Darmor-*bzh* × Yudal DH lines

To map QTL for resistance in the pre-existing Darmor-*bzh* × Yudal DH mapping population (Delourme et al. 2006), a new genetic map was generated using a *B. napus* 20 K SNP array. 8152 high-quality polymorphic SNPs were identified and assembled into an initial genetic map representing the 19 *B. napus* linkage groups. This map was simplified to a minimal map of the 1298 markers required to distinguish all the recombination events in the population and annotated according to their corresponding physical chromosomes in the *B. napus* genome (Chr A01 to A10 and Chr C01 to C09). The overall size of the linkage map was 2196.3 cM with minimum spacing of 1.7 cM and maximum spacing of 13.3 cM. The smallest linkage group was Chr A04 (65 cM) with 39 markers and the largest was Chr C03 (196.6 cM) with 114 markers (Supp Fig. 2).

**Fig. 2:**
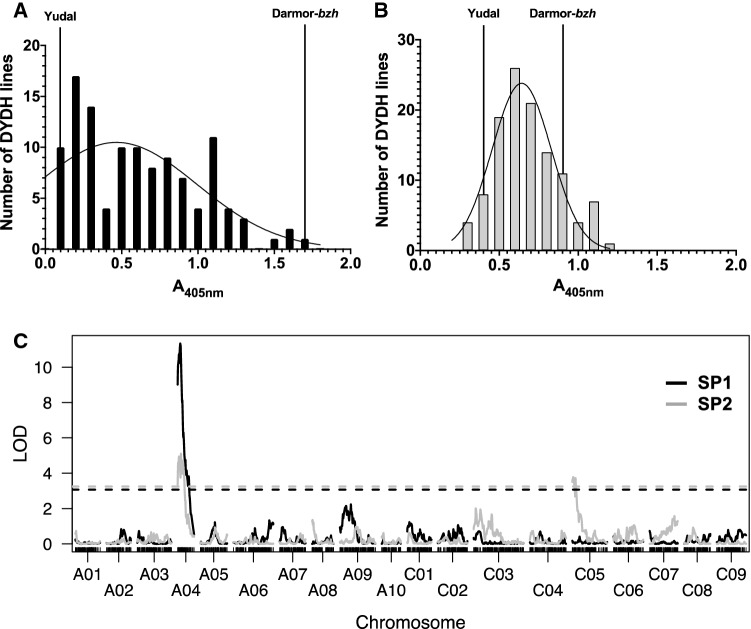
Frequency distribution of Turnip yellows virus titres in Yudal x Darmor-*bzh* doubled-haploid (DYDH) lines in experiments SP1 (**a**) and SP2 (**b**). Virus titres of the resistant (Yudal) and susceptible (Darmor-*bzh*) parents and nonlinear regression of a Gaussian distribution curve are indicated. **c** LOD plot of QTL analysis of DH populations SP1 experiment 1 using non-parametric interval mapping (black) and SP2 experiment 2 using Haley & Knott (grey). LOD threshold (α ≤ 0.05) of 10,000 permutations is indicated as a dashed line

TuYV titres in 115 DYDH lines were determined in two independent experiments (SP1 and SP2). Overall mean ELISA values of both experiments were similar (0.62 for SP1 and 0.68 for SP2) and both test populations showed significant positive correlation in Spearman correlation test (*r*_*s*_ = 0.3808, *P* < 0.0001, *α* = 0.05). Experiment SP1 and SP2 differed in their overall range of absorbance values (0.09–1.73 for SP1 and 0.27–1.18 for SP2); the majority of DH lines in SP1 and SP2 (93 and 79%, respectively) showed mean virus titres intermediate between the resistant and susceptible parental means (Fig. 2a, b). None of the test populations showed a clear skewed or bimodal distribution (median_SP1_ = 0.6 and median_SP2_ = 0.7).

The distribution of ELISA values in SP1 (Fig. 2a) deviated significantly from a normal distribution. Appropriate transformation of SP1 ELISA values did not result in significant normality. Non-parametric QTL analysis (Fig. 2c, Table 2) detected one significant QTL (qTUYVA4) with a LOD score of 11.3, explaining 36.01% of the phenotypic variation at position 10 cM on linkage group Chr A04. The QTL 1.5 LOD confidence interval (CI) was flanked by marker scaffoldv4_71_1383505 at position 0 cM and scaffoldv4_649_37158 at position 13.9 cM, comprising a 13.9 cM QTL region (Fig. 4).

**Table 2:** Details of quantitative trait loci (QTL) for Turnip yellows virus (TuYV) resistance detected in a BC_1_ population (Darmor-*bzh* × [Yudal × DYDH130]) and in two experiments (SP1 and SP2) on the DYDH population

Ex.	Name	Chr	Pos (cM)	CI	LOD (*α* < 0.05)	LOD	% *R*^2^	Additive effect	*df*	Flanking markers
SP1	qTUYVA4	A04	10	0–14	3.07	11.3	36.0	− 0.5226	114	scaffoldv4_71_1383505
										scaffoldv4_649_37158
SP2	qTUYVA4	A04	12.9	0–27	3.25	5.11	18.2	− 0.1637	114	scaffoldv4_71_1383505
										scaffoldv4_455_425140
	qTUYVC5	C05	3	0–19	3.25	3.78	13.8	0.1388	114	scaffoldv4_266_34073
										scaffoldv4_215_634572
BC_1_	qTUYVA4	A04	15.5	0–21	2.91	2.95	11.9	− 0.2318	106	Bn-A04-p560622
										Bn-A04-p10088142

ELISA values in SP2 were normally distributed (Fig. 2b) and single QTL analysis (Fig. 2c, Table 1), multiple QTL modelling (Supp Table 1) and pairwise QTL analysis (Supp Table 2) identified two QTLs, one on Chr A04 and the other on Chr C05, which act additively, without interaction, according to the QTL model with the highest LOD score. The results for SP2 confirmed qTUYVA4 on Chr A04 as detected in SP1, although with a lower LOD score (LOD = 5.11, LOD_threshold_ = 3.25; *α* < 0.05) and a wider 1.5 LOD interval, spanning a region of 27 cM between marker scaffoldv4_71_1383505 and scaffoldv4_455_425140 (Fig. 4). The QTL on Chr C05 (LOD = 3.78, LOD_threshold_ = 3.25; *α* < 0.05) explained slightly less of the phenotypic variation than that of Chr A04 (13.8% vs. 18.2%) and comprised a 1.5 LOD confidence interval between positions 0 cM and 19 cM.

### QTL analysis for TuYV resistance in the BC_1_ population

**Fig. 3:**
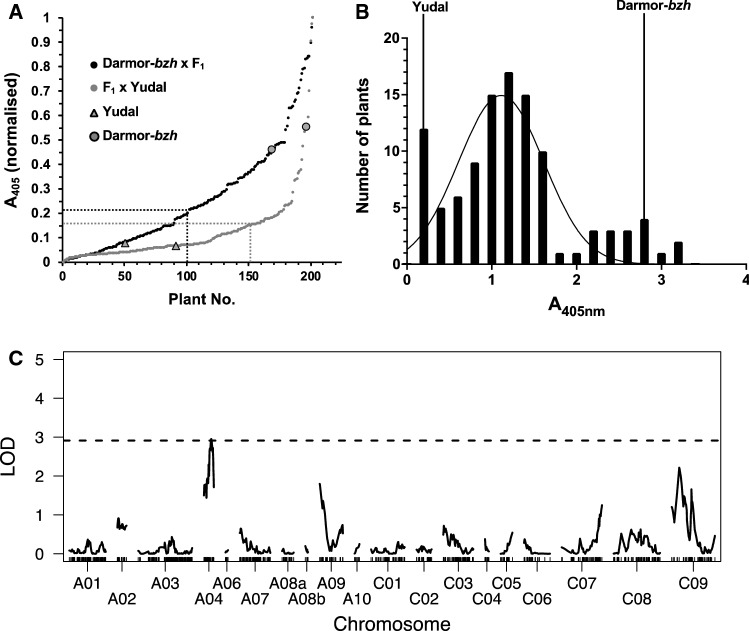
**a** Ranked Turnip yellows virus (TuYV) titres after normalisation to the maximum and minimum TAS-ELISA absorbance values (A_405_) for the BC_1_ populations Darmor-*bzh* × [Yudal × DYDH130] (black) and [Yudal × DYDH130] × Yudal (grey) together with mean values of 10–20 Yudal (triangle) and Darmor-*bzh* (circle) control plants. The mean values for Yudal + standard deviation (threshold_Yudal+SD_) for each of the BC_1_ populations are indicated as a dashed line. **b** Frequency distribution of TuYV titres in the Darmor-*bzh* × [Yudal × DYDH130] BC_1_ population. Mean values of 10 resistant (Yudal) and susceptible (Darmor-*bzh*) control plants and nonlinear regression of a Gaussian distribution curve are indicated. **c** LOD plot of QTL analysis of BC_1_ population (Darmor-*bzh* × [Yudal × DYDH130]) using Haley & Knott. LOD threshold (*α* ≤ 0.05) of 10,000 permutations is indicated as dashed line

To determine dominance/recessivity, further investigate the potential QTL identified in the DH population and attempt to increase the mapping resolution, BC_1_ populations were explored. To generate a parental cross, one of the TuYV-susceptible DYDH lines (DYDH130) was crossed with Yudal (Yudal × DYDH130). The rationale for crossing a susceptible line to the resistant parent was to ensure all potential Yudal resistance alleles that would be represented in the segregating BC_1_ population which might not have been the case if a resistant DYDH line was crossed with Darmor-*bzh*. F_1_ plants from this cross were used to generate two BC_1_ populations by crossing with the susceptible Darmor-*bzh* as female (Darmor-*bzh* × [Yudal × DYDH130]) and with the partially resistant Yudal as male parent ([Yudal × DYDH130] × Yudal; Supp Fig. 1). Two-hundred plants of each BC_1_ population were challenged with TuYV and virus titres were determined (Fig. 3a). Both BC_1_ populations showed a continuous distribution of ELISA values. Of the BC_1_ plants from the [Yudal × DYDH130] × Yudal cross, 79.5% showed ELISA values lower than the mean ELISA value of the Yudal control plants + standard deviation (threshold_Yudal+SD_). In contrast, of the BC_1_ plants from the Darmor-*bzh* × [Yudal × DYDH130] cross, 50.5% had ELISA values lower than the threshold_Yudal+SD_. As the backcross to the resistant parent ([Yudal × DYDH130] × Yudal) resulted in a vast majority of BC_1_ partially resistant individuals, whilst the backcross with the susceptible parent (Darmor-*bzh* × [Yudal × DYDH130]) showed segregation of TuYV-partially resistant and TuYV -susceptible BC_1_ individuals, this indicated that the resistance in Yudal is dominant, but only partially dominant.

A subgroup of 107 plants of the segregating BC_1_ population from the Darmor-*bzh* × [Yudal × DYDH130] cross representing the range of the measured virus titres was genotyped for QTL analysis. Polymorphic SNPs (7790) were obtained and assembled into a minimal genetic map of 567 markers as described above for the DYDH map. Markers in this map only segregate in regions of the genome where the DYDH130 has the Darmor-*bzh* genotype and so were not informative for QTL analysis in the DYDH130 Yudal genome segments where all the genotypes in the BC_1_ population would be heterozygous. The overall map size was 934.3 cM, and minimum and maximum spacing was 1.7 cM and 18.9 cM, respectively. The linkage groups were annotated in accordance with the physical chromosomes. However, no linkage group representing ChrA05 was identified because this was entirely Yudal genotype in DYDH130, although this was not known at the time of generating the crosses. ChrA08 was annotated as two linkage groups named ChrA08A and B again because this linkage group had two Darmor-*bzh* segments separated by a Yudal segment. The linkage groups ranged from 5.6 cM and 6 markers (ChrA06) to 115.8 cM and 79 markers in ChrA03 (Supp Fig. 3).

**Fig. 4:**
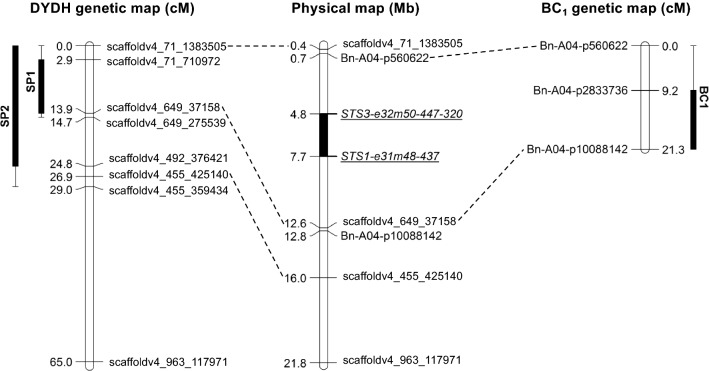
Genetic linkage maps of ChrA04 from DYDH and BC_1_ experiments and physical position of Turnip yellows virus (TuYV) resistance QTL on ChrA04 using the *Brassica rapa* genome assembly GCA_000309985.2 v3.0

Following TuYV challenge, the mean viral titre (A_405nm_) of this BC_1_ population was 1.25 and ranged from 0.19 to 3.3 (Fig. 3b). The ELISA values of the Darmor-*bzh* × F_1_ BC_1_ population had to be transformed ($\sqrt {\text{A}_{405{\text{nm}}}}$) to obtain normal distribution. Single QTL analysis (Fig. 3c; Table 2) and multiple QTL modelling (Supp Table 1) identified a single QTL on linkage group ChrA04, confirming qTUYVA4 in the BC_1_ population. The position of the QTL was determined at 15.5 cM with a LOD score of 2.95, slightly above the genome-wide significance threshold of 2.91 LOD (*α* < 0.05), explaining 11.9% of the phenotypic variation. The QTL was linked to markers Bn-A04-p560622 at position 0 cM (LOD = 1.50) and Bn-A04-p10088142 at position 21.3 cM (LOD = 1.71), comprising the entire linkage group (Fig. 4). No other significant QTL was detected.

### Physical position of TuYV resistance in Yudal

The physical position of the qTUYVA4 flanking markers, identified in each of the DYDH and BC_1_ experiments, was determined by sequence comparison with the *B. napus* genome of Darmor-*bzh* (assembly: GCA_000751015.1) (Chalhoub et al. 2014) using the BLASTN algorithm (Altschul et al. 1990). In all the three experiments, the qTUYVA4 1.5 LOD intervals comprised overlapping regions of Chr A04. The smallest interval was found in experiment SP1 (10.75 Mb) and was entirely enclosed in the larger intervals identified in the SP2 and BC_1_ experiments (13.79 Mb and 10.79 Mb). The minimal qTUYVA4 interval comprises 1262 annotated genes in *B. napus*. Since the QTL associated with TuYV resistance in ‘R54’ was also mapped on Chr A04, we compared its physical position with that of qTUYVA4. In the original Darmor-*bzh B. napus* genome assembly (Chalhoub et al. 2014), none of the co-segregating markers for the TuYV resistance in ‘R54’ (STS3e32m50-447-320 and STS1e31m48-437) were located on Chr A04, but could be identified on a non-annotated chromosomal scaffold (ChrAnn_random). However, both ‘R54’ TuYV resistance markers were unambiguously found on Chr A04 of the re-annotated Darmor-*bzh B. napus* genome assembly v8.1 (Bayer et al. 2017) and the *B. rapa* genome assembly (GCA_000309985.2) (Wang et al. 2011), encompassing approximately 3 and 4.7 Mb regions, respectively, located inside the qTUYVA4 interval (Fig. 4). PCR analysis using the ‘R54’ TuYV resistance molecular markers, suggested the possible absence of the ‘R54’ resistance-linked alleles in Yudal and Darmor-*bzh*. Sanger sequencing of the amplicons for STS3e32m50-447-320 and STS1e31m48-437 (385 bp and 376 bp, respectively) revealed identical sequences of both markers in Yudal and Darmor-*bzh*, possessing the size of those from the susceptible plants described in the mapping of the resistance in ‘R54’; STS3e32m50-447-320 was 41 bp larger and STS1e31m48-437 was 61 bp smaller than ‘R54’ (Juergens et al. 2010). In contrast, the *B. napus* cultivar ‘Caletta’ (possesses the ‘R54’ TuYV resistance) showed homozygosity for the co-segregating alleles of the ‘R54’ TuYV resistance (344 bp for STS3e32m50-447-320 and 437 bp for STS1e31m48-437).

## Discussion

### TuYV resistance in *B. napus*

TuYV is one of the most widespread and common diseases, causing severe yield losses in commercial oilseed rape and vegetable brassica crops in Europe. To date, no brassica germplasm with complete immunity to TuYV has been reported. The only mapped genetic resource for TuYV resistance in brassica is the incomplete resistance in the re-synthesised *B. napus* line ‘R54’ (Graichen 1994; Juergens et al. 2010), which is thought to have derived from its parental A-genome donor Chinese cabbage (Dreyer et al. 2001). In this study, we report for the first time the occurrence of TuYV resistance in a natural allotetraploid *B. napus* variety. Although the resistance was incomplete, TuYV titres in Yudal were 1.7–4.1 times lower than in any other tested accession. The resistance appears to be commercially useful, as in a controlled experiment, TuYV-challenged Yudal did not have a significantly lower seed yield than unchallenged Yudal controls, whereas other commercial varieties had significantly reduced yields (Asare-Bediako 2011).

### TuYV resistance inheritance and QTLs

One major QTL for TuYV resistance in Yudal was found on ChrA04 (qTUYVA4). It was significantly associated with TuYV resistance and explained between 18 and 36% of the phenotypic variation. We determined the dominance of the TuYV resistance by phenotyping two BC_1_ populations.

However, the segregation of virus titres in these BC_1_ populations (Fig. 3a) indicated that the resistance was actually partially dominant. The qTUYVA4 interval identified in the DYDH population was verified in the segregating BC_1_ population, albeit with a low LOD score of only 2.95, just passing the genome-wide significance threshold. The LOD scores for qTUYVA4 in the homozygous DYDH population were 11.3 and 5.1 in SP1 and SP2, respectively, exceeding the genome-wide significance thresholds by far and the corresponding 1.5 LOD confidence interval narrowed qTUYVA4 down to 14/10.8 and 27/13.9 cM/Mb regions on Chr A04. In the segregating BC_1_ population, the qTUYVA4 confidence interval comprised the entire ChrA04 linkage group, providing no further resolution of qTUYVA4. This was mainly due to the weak association of qTUYVA4 with TuYV resistance and the low recombination frequency on Chr A04 in the BC_1_. According to the minimal spanning tree of the BC_1_ genetic map, only 19 markers covered all recombination events on the 21 cM/12.8 Mb segment of Chr A04 (Supp Fig. 3) inherited from DYDH130, representing only 58% of the physical map of Chr A04 (22.5 Mb). In contrast, the DYDH genetic map covers 21.8 Mb of Chr A04 generating a 65 cM linkage group for Chr A04 and enabled mapping of qTUYVA4 between markers at positions 0.4 and 12.6 Mb (Fig. 4). The weak association of qTUYVA4 with TuYV resistance in the BC_1_ population is most likely a consequence of the heterozygosity of TuYV resistance gene(s) in the BC_1_ and the ability to only phenotype single plants. In contrast, QTL mapping using the homozygous DH population provided higher LOD scores and resolution and facilitated phenotyping of several plants for each DH line in multiple experiments. This demonstrates that a DH mapping strategy was clearly advantageous for mapping this quantitative virus resistance trait.

Consistent with the nature of a quantitative trait, TuYV titres measured using the TAS-ELISA technique resulted in continuous distributions of phenotypes in all DYDH and BC_1_ experiments. None of the DYDH and BC_1_ phenotyping experiments showed a clear bimodal distribution of susceptible to resistant plants, as would be predicted for a 1:1 segregation of a strong monogenic trait. Thus, TuYV resistance in Yudal may depend on additional contributing genes, environmental factors and/or may be an artefact of the phenotyping of this partially dominant trait. The mapping of TuYV resistance in ‘R54’ showed a clear single QTL, but was based on phenotypes also not showing a bimodal distribution (Dreyer et al. 2001). Nevertheless, the markers derived from this mapping approach have been very successful in introgressing the resistance from ‘R54’ into the commercial oilseed rape variety ‘Caletta’ (Graichen and Peterka 1999). A second QTL qTUYVC5 was identified in one of the DH experiments (SP2), explaining 11.9% of the phenotypic variation, acting additively, without interaction with qTUYVA4. The estimated effects of the QTLs on Chr A04 and Chr C05 (Table 2) showed opposite effects of allelic substitution, suggesting that both parental lines contributed to the resistance response. Although susceptible to TuYV, Darmor-*bzh* did not show the highest virus titre amongst the 27 tested *B. napus* accessions. However, as the qTUYVC5 effect was only seen in one of the two experiments on the DYDH lines, it remains speculative as to whether the slightly lower TuYV infection in Darmor-*bzh* was actually caused by genetic factors. No additional significant QTL were detected in either the DYDH experiment SP1 or in the segregating BC_1_ population, suggesting that the contribution of qTUYVC5 towards TuYV resistance may have been due to specific environmental conditions present during the SP2 experiment. The promoting effect of increased temperature on TuYV susceptibility in ‘R54’ and oilseed rape varieties was described previously (Dreyer et al. 2001; Graichen 1998). However, SP1 and SP2 were carried out as replicated glasshouse experiments under similar temperature regimes, but during different seasons (autumn and winter). Additional factors like light intensity or day length could have played a role during the complex interaction between plants, aphids and TuYV, influencing the course of virus infection and ultimately eliciting additional genes to contribute towards the interactions.

TuYV resistance in Yudal and in ‘R54’ are both associated with one major QTL, located on Chr A04. The genomic region between the two co-segregating markers of the ‘R54’ QTL is within the larger 10.75 Mb CI of qTUYVA4. The ‘R54’ QTL and qTUYVA4 share about 166 genes. However, qTUYVA4 includes 1096 additional gene loci, which are not co-segregating with the ‘R54’ TuYV QTL, representing a plethora of potential alternative candidate loci for the TuYV resistance in Yudal. Intriguingly, both TuYV resistance sources are dominant and quantitative. As the co-segregating markers for the TuYV resistance in ‘R54’ are conserved in the commercial variety ‘Caletta’ (possessing resistance derived from ‘R54’), but are not conserved in Yudal, this could indicate that the resistance alleles in ‘R54’ and qTUYVA4 are not derived from the same source. This suggests that both *B. napus* lines are very likely to have originated from different *B. rapa* A-genome donors. Yudal (*B. napus* var. *oleifera*) is a native rapeseed inbred line of Korean origin (Jeong et al. 2012; Wagner et al. 2019), developed as a high erucic acid *Brassica napus* cultivar in 1969 (Choy et al. 2007; Kae et al. 1971). As a native allotetraploid *B. napus* variety, Yudal’s A-genome presumably derived from the natural hybridisation of *B. rapa* and *B. oleracea*, ~ 5000–10,000 years ago. In contrast, ‘R54’ was generated via re-synthesis in the 1970s at the University of Göttingen (Juergens 2009), using a cross between the cabbage cultivar ‘Stone Head’ (*B. oleracea* var*. capitata*) and the Chinese cabbage ‘No.67’ (*B. rapa* ssp. *pekinensis*). As the underlying molecular mechanism and the gene(s) involved in the TuYV resistances in ‘R54’ and Yudal are unknown, it is not feasible to dismiss the possibility that both resistances are based on similar genetic loci.

TuYV infection and TuYV-related yield losses are predicted to become more severe in the future as a consequence of increasing temperature due to climate change. There are currently a number of commercial varieties of OSR with partial resistance to TuYV (for example, Amalie, Aspire, Annalise, Architect, Ambassador, Artemis, Aurelia from Limagrain, Saint-Beauzire, France; Darling, Dazzler, Ludger, Temptation from DSV, Lippstadt, Germany; Allessandro, Feliciano from KWS, Einbeck, Germany; Atora, Dominator from Rapool-Ring, Isernhagen, Germany; Cadran, Coogan from RAGT, Rodez, France; Addition from Soufflet Seeds, Poznań, Poland; DMH440 from Dekalb AgResearch, Dekalb, USA). Many of these possess resistance derived from ‘R54’ and no other independent TuYV resistance source is described for any of these varieties. Growing these varieties is creating strong selection pressure for resistance-breaking strains of TuYV. Additional independent sources of resistance are, therefore, essential to reduce selection pressure for ‘R54’ resistance-breaking TuYV and promote durable control of the virus in the future. It remains to be seen whether Yudal will provide a useful additional source of resistance to TuYV.

## Electronic supplementary material

Below is the link to the electronic supplementary material.

**Supplementary Table** **1**: Multiple QTL modelling for DYDH SP2 and Darmor-*bzh* x [Yudal x xDYDH130] BC_1_. **Supplementary Table** **2**: Permutation-based testing for DYDH SP2 based on an assumption. 2-QTL model (scantwo), accounting for potential additive and epistatic effects. **Supplementary Fig.** **1**: Schematic overview of crosses used in this study. **Supplementary Fig.** **2**: Genetic linkage map of the *Brassica napus* genome for the DYDH population. **Supplementary Fig.** **3**: Genetic linkage map of the *Brassica napus* genome for the Darmor-*bzh* x [Yudal x DYDH130] BC_1_ population (PDF 9209 kb)
