# Supplementary material for: Identification and QTL mapping of resistance to Turnip yellows virus (TuYV) in oilseed rape, Brassica napus
Source: Theor Appl Genet. 2019 Nov 5;133(2):383–93. doi: 10.1007/s00122-019-03469-z (PMC6985063; doi:10.1007/s00122-019-03469-z)
Supplement: Supplementary file 1 — Supplementary Table 1: Multiple QTL modelling for DYDH SP2 and Darmor-bzh x [Yudal x xDYDH130] BC1. Supplementary Table 2: Permutation-based testing for DYDH SP2 based on an assumption. 2-QTL model (scantwo), accounting for potential additive and epistatic effects. Supplementary Fig. 1: Schematic overview of crosses used in this study. Supplementary Fig. 2: Genetic linkage map of the Brassica napus genome for the DYDH population. Supplementary Fig. 3: Genetic linkage map of the Brassica napus genome for the Darmor-bzh x [Yudal x DYDH130] BC1 population (PDF 9209 kb) [file 122_2019_3469_MOESM1_ESM.pdf]

Supplementary Table 1 Multiple QTL modelling for DYDH SP2 and Darmor-*bzh* x [Yudal x xDYDH130] BC_1. Interacting QTLs are in brackets and models with the highest LOD are highlighted in grey.

Experiment	model	pLOD
SP2	A04[12.9cM]	2.42
	**A04[12.9cM]+C05[2cM]**	**3.56**
	A04[12.9cM]+C05[2cM]+C03[71.5cM]	2.98
	(A04[12.9cM]+C08[35.4cM])+C05[1cM]+C03[26.9cM]	2.44
BC_1	**A04[15.5cM]**	**0.26**
	A04[15cM]+C09[16.1cM]	-0.55
	(A04[15cM]+C05[16.1cM])	-1.36
	(A04[15.5cM]+C09[16.1cM])+A03[45cM]	-2.7
	(A04[15.5cM]+C09[16.1cM])+A03[44.7cM]+A09[0cM]	-4.14

Supplementary Table 2 Permutation-based testing for DYDH SP2 based on an assumed 2-QTL model (scantwo), accounting for potential additive and epistatic effects. Likely model is highlighted (grey).

Assumed model	LOD score/significance threshold for any 2 loci (based on 1,000 permutations, α = 0.05)	LOD score for **A04**[13] + **C05**[2]
Mf	7.06	8.89
Ma	**5.55**	**8.88**
Mi	4.93	5.38×10^{-3}
Mfv1	5.53	3.84
Mav1	3.38	3.84

Mf, full model; *Ma*, additive model; *Mi*, interactive effects; *Mfv1*; full model versus a single QTL model; *Mav1*; additive model versus a single QTL model.

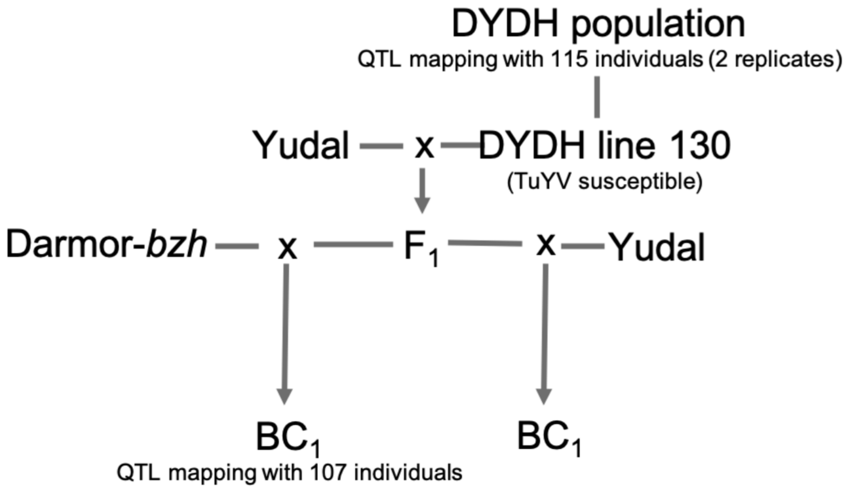

Supplementary Fig. 1 Schematic overview of crosses used in this study

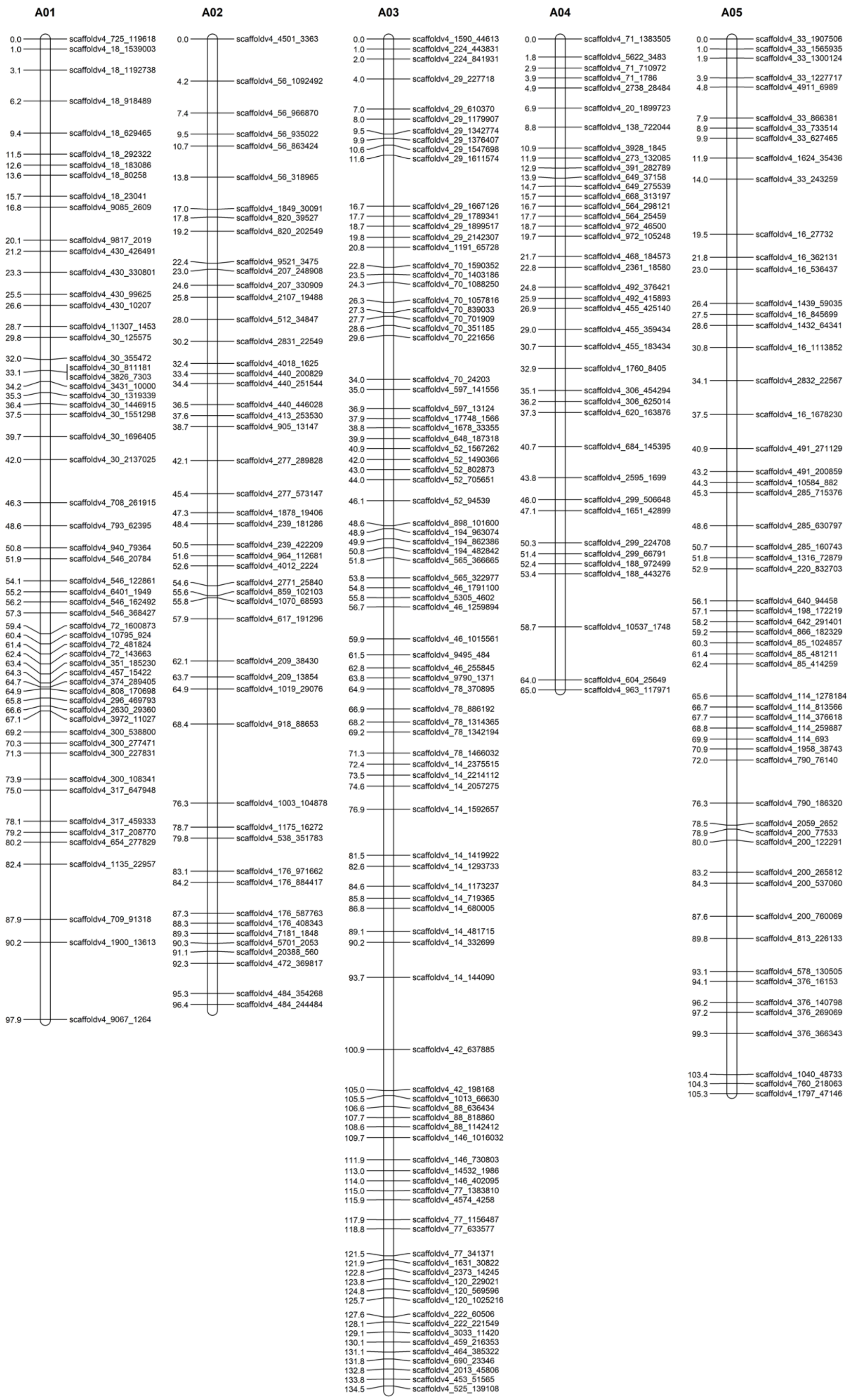

Supplementary Fig. 2 Genetic linkage map of the *Brassica napus* genome for the DYDH population

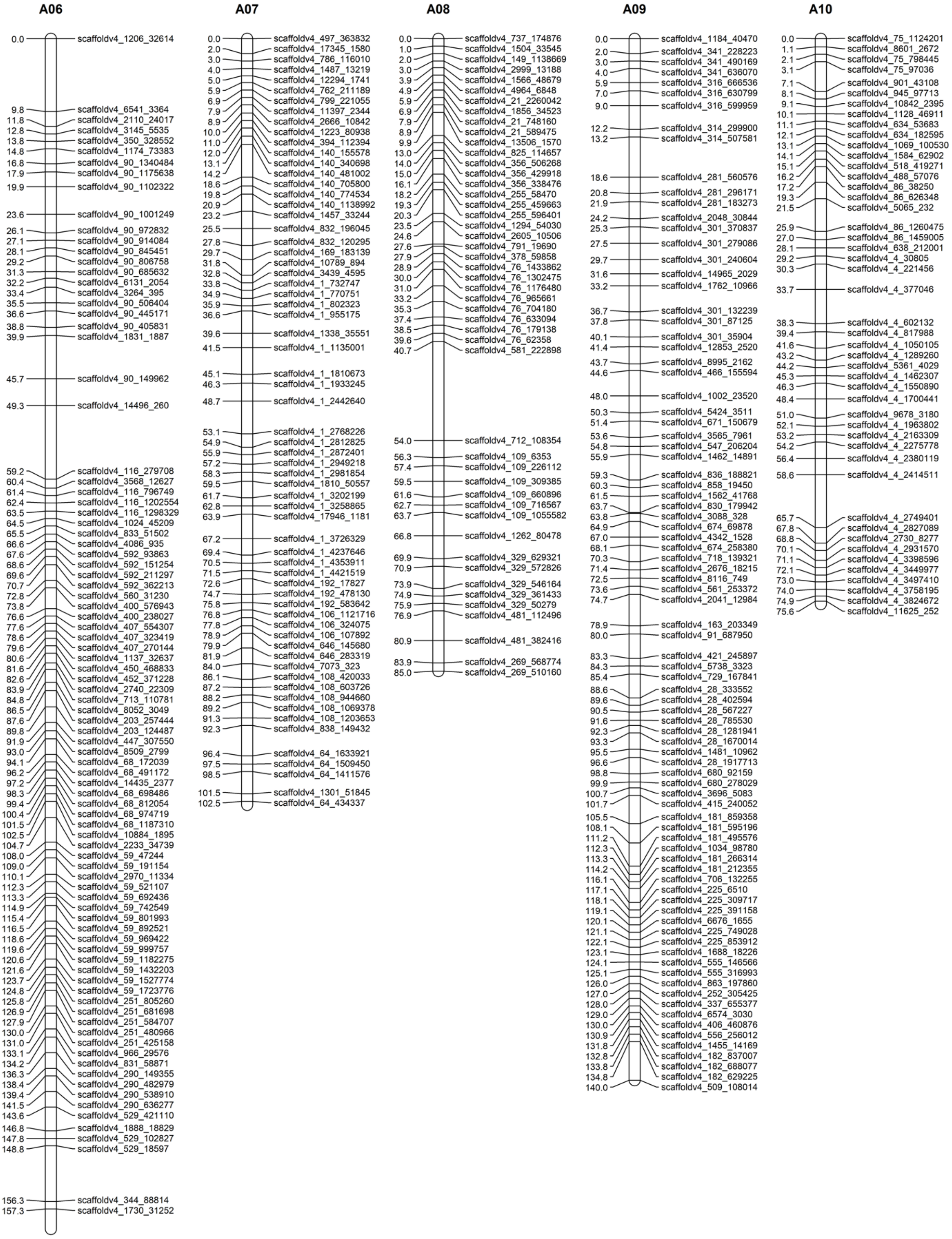

Supplementary Fig. 2 (continued) Genetic linkage map of the *Brassica napus* genome for the DYDH population

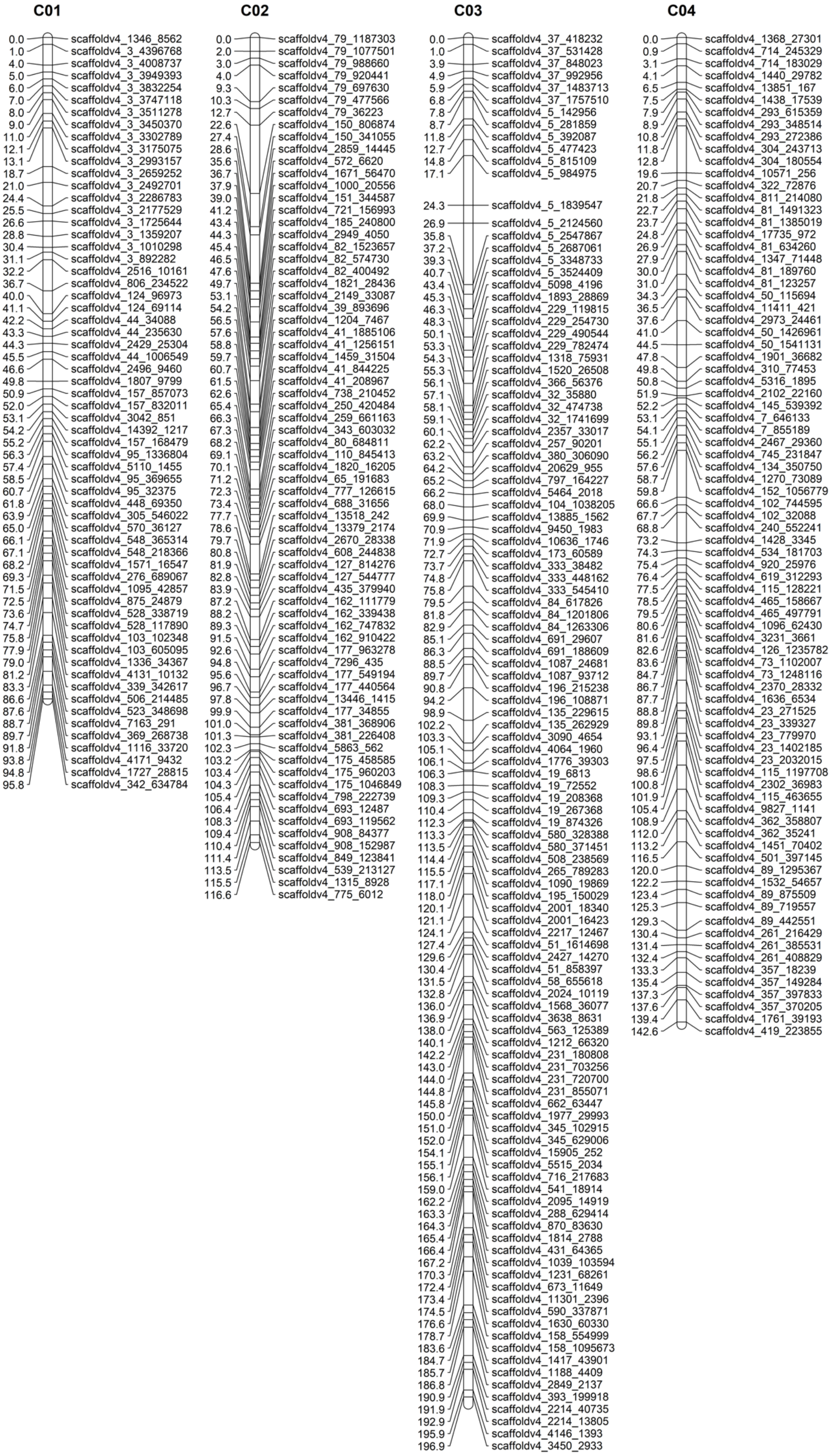

Supplementary Fig. 2 (continued) Genetic linkage map of the *Brassica napus* genome for the DYDH population

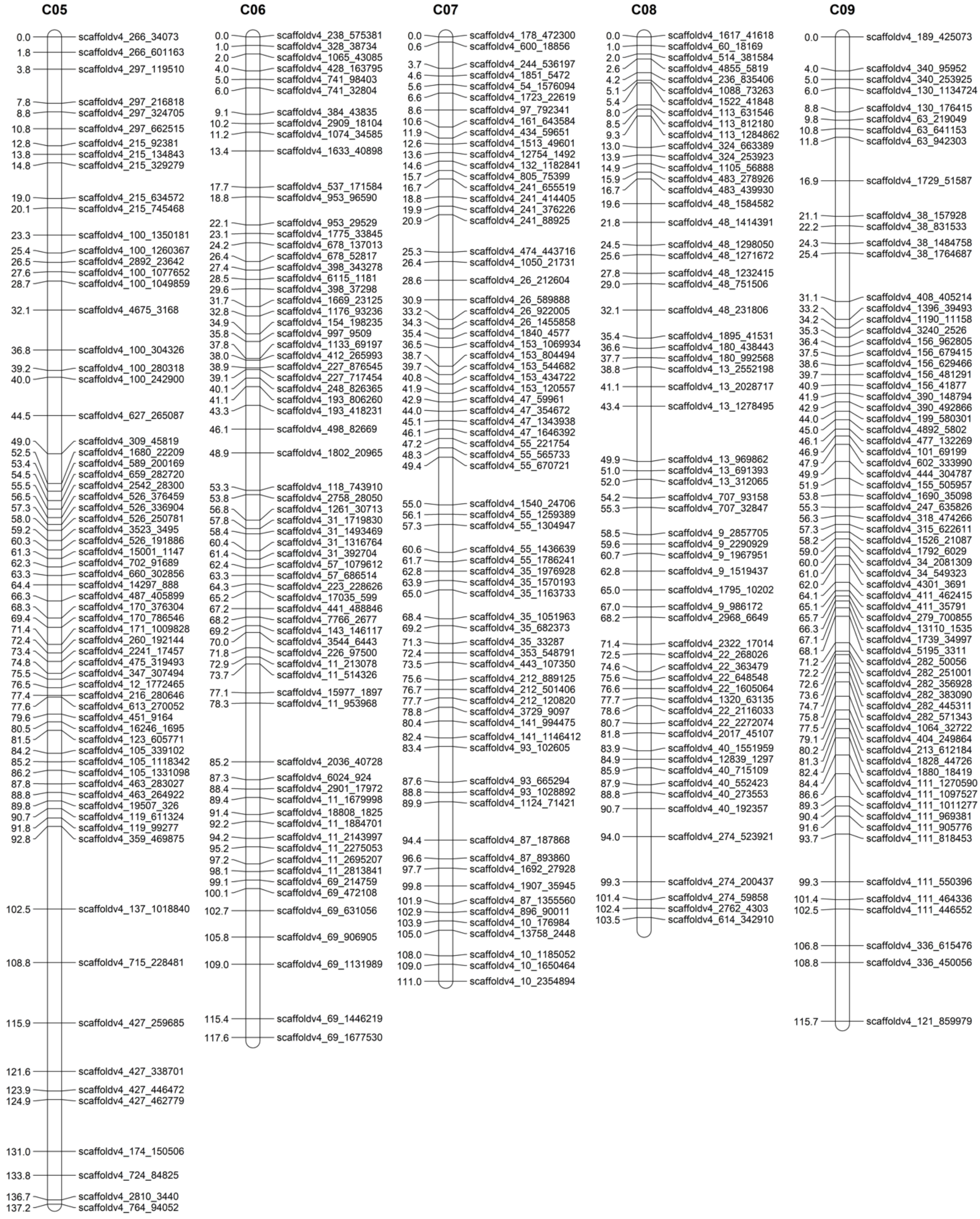

Supplementary Fig. 2 (continued) Genetic linkage map of the *Brassica napus* genome for the DYDH population

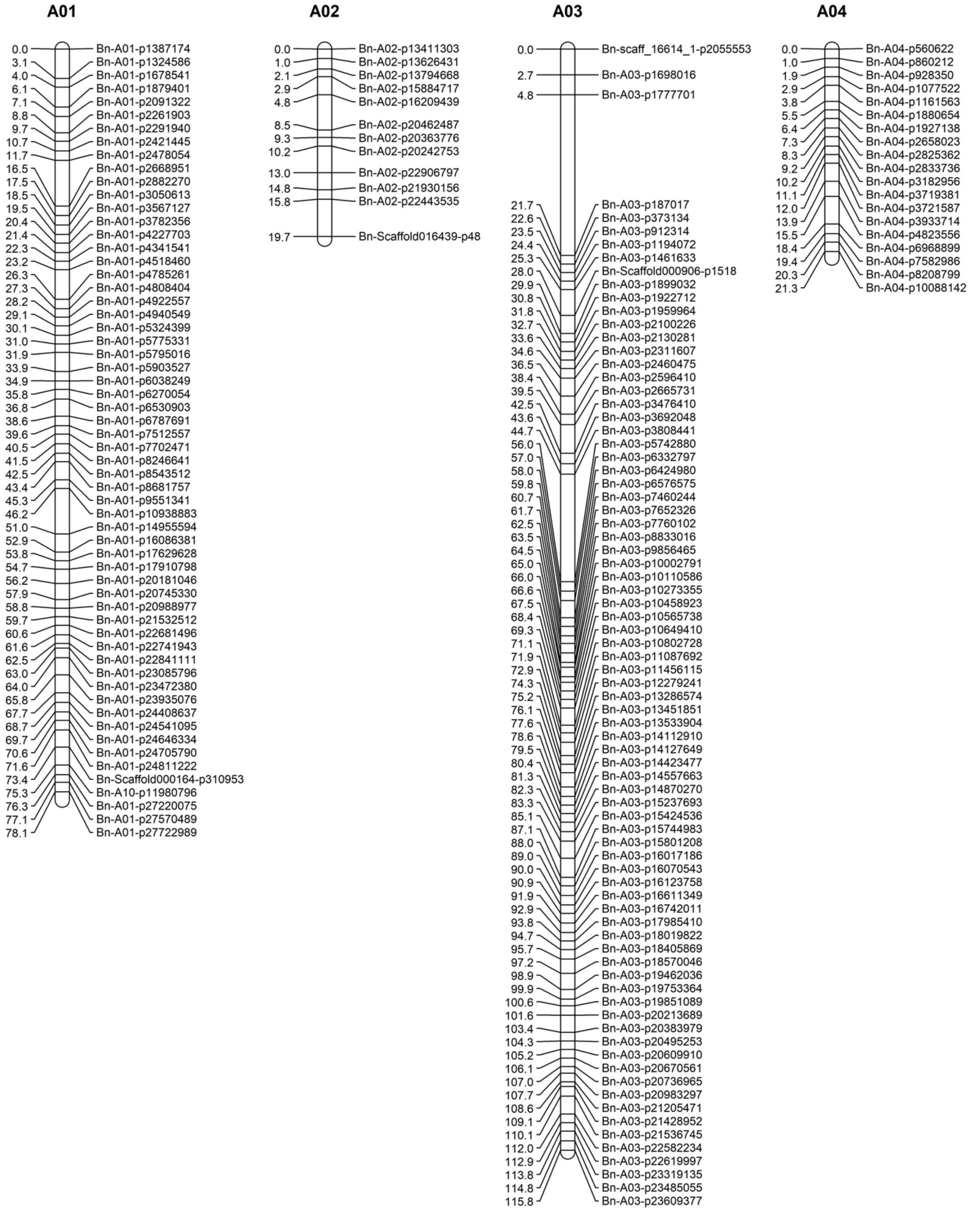

Supplementary Fig. 3 Genetic linkage map of the *Brassica napus* genome for the Darmor-*bzh* x [Yudal x DYDH130] BC_1 population

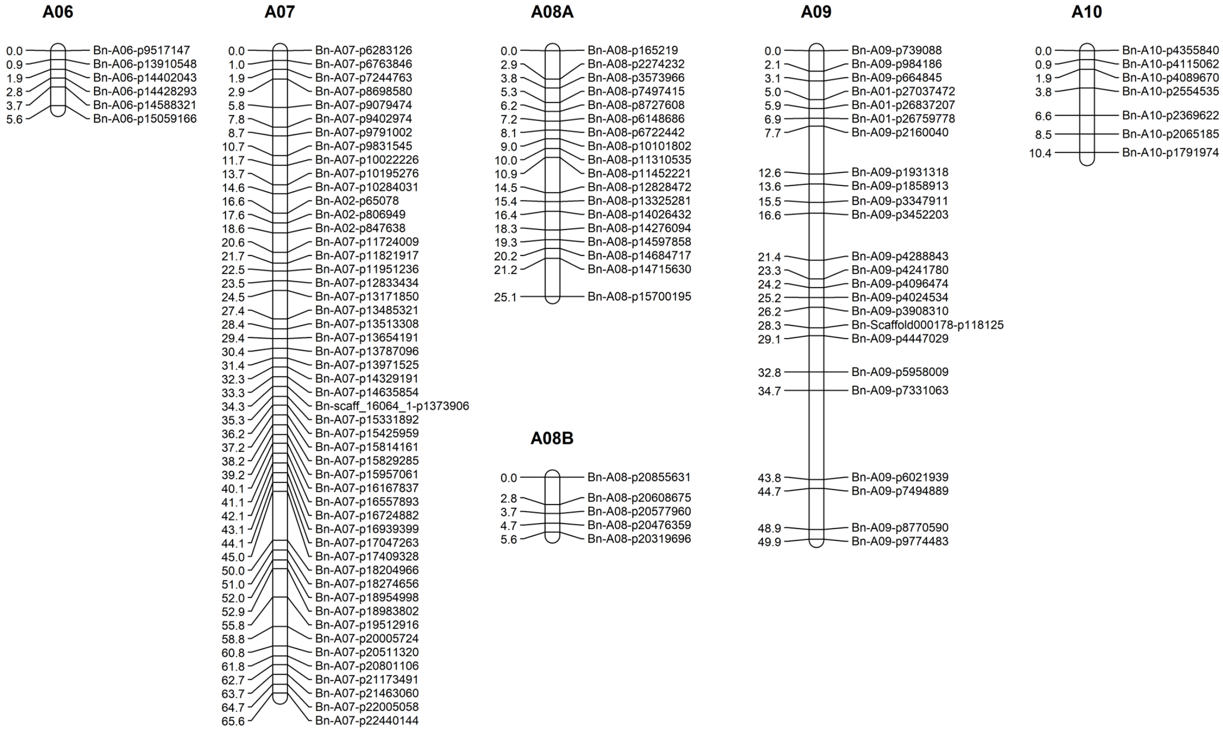

Supplementary Fig. 3 (continued) Genetic linkage map of the *Brassica napus* genome for the Darmor-*bzh* x [Yudal x DYDH130] BC_1 population

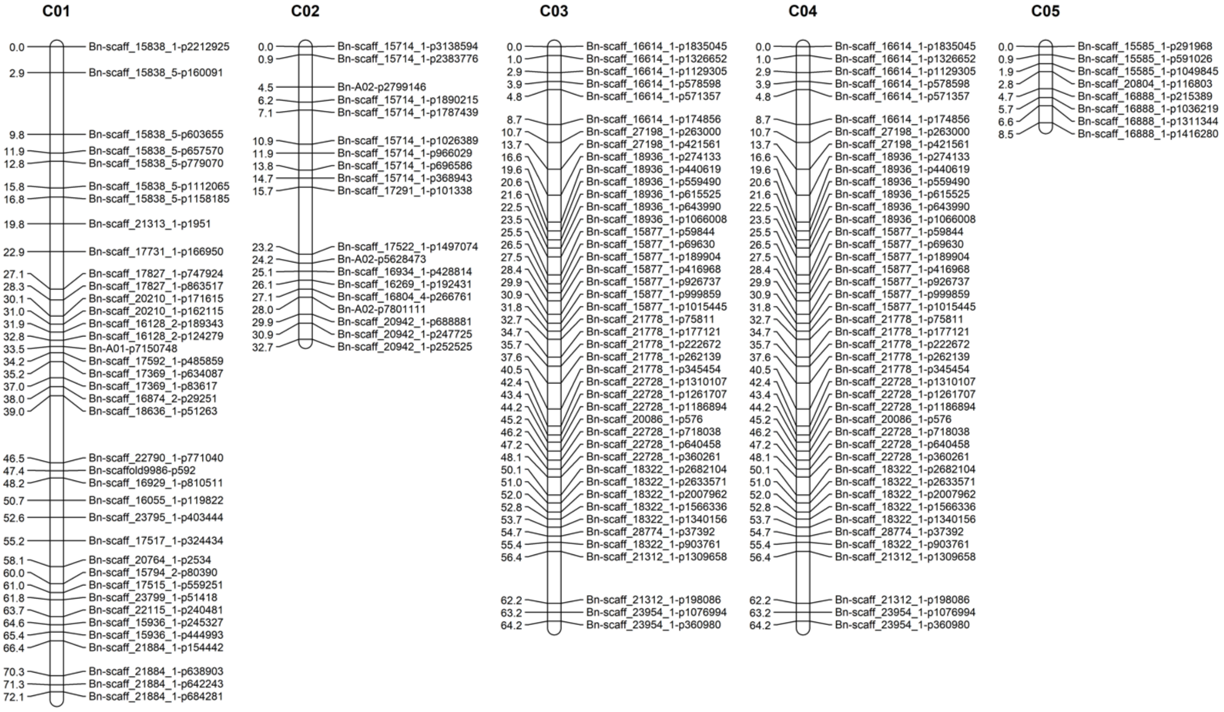

Supplementary Fig. 3 (continued) Genetic linkage map of the *Brassica napus* genome for the Darmor-*bzh* x [Yudal x DYDH130] BC_1 population

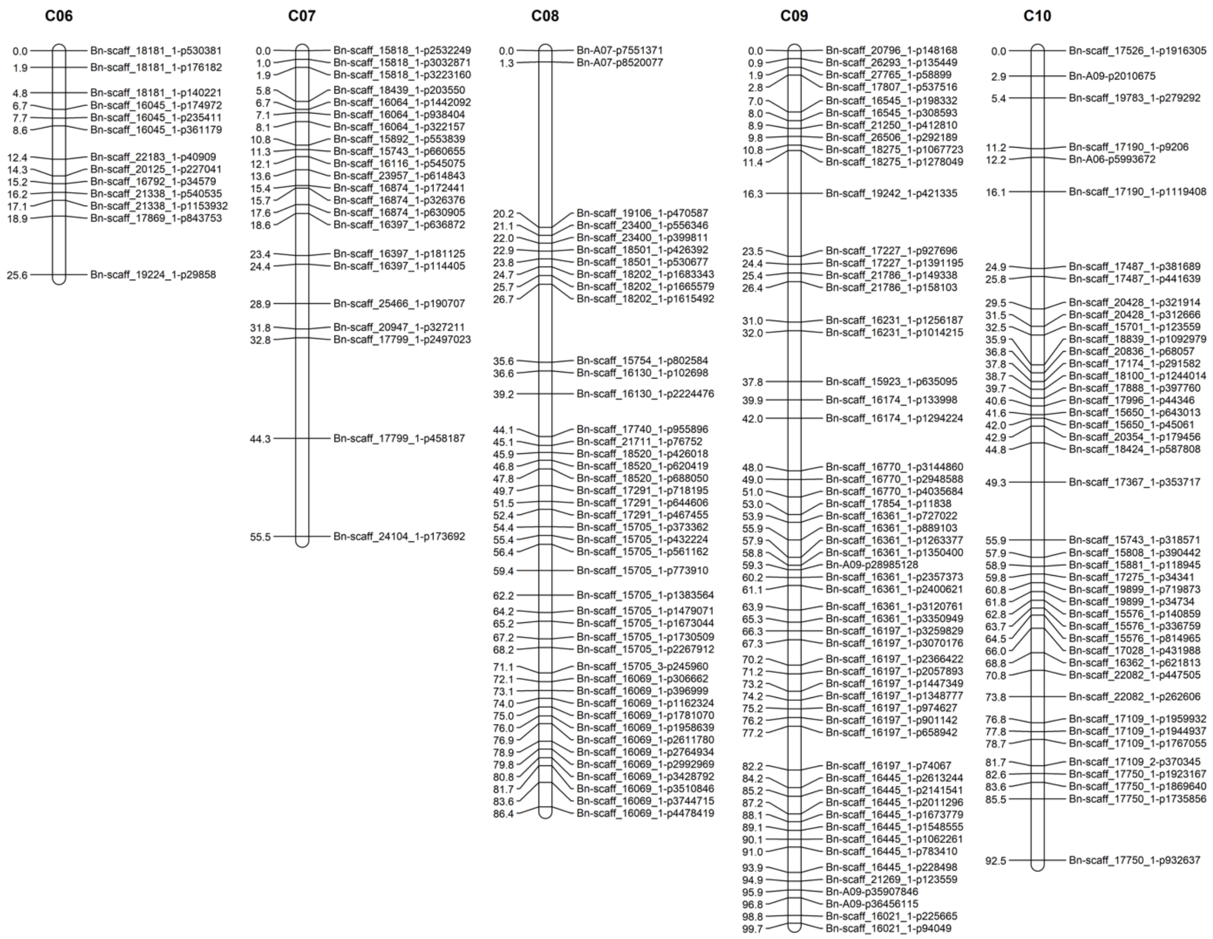

Supplementary Fig. 3 (continued) Genetic linkage map of the *Brassica napus* genome for the Darmor-*bzh* x [Yudal x DYDH130] BC_1 population
